# Critical Contribution of NK Group 2 Member D Expressed on Invariant Natural Killer T Cells in Concanavalin A-Induced Liver Hepatitis in Mice

**DOI:** 10.3389/fimmu.2018.01052

**Published:** 2018-05-17

**Authors:** Dina Al Dulaimi, Jihene Klibi, Veronica Olivo Pimentel, Veronique Parietti, Matthieu Allez, Antoine Toubert, Kamel Benlagha

**Affiliations:** ^1^INSERM, UMR-1160, Institut Universitaire d’Hématologie, Paris, France; ^2^Université Paris Diderot, Sorbonne Paris Cité, Paris, France; ^3^Département d’Expérimentation Animale, Institut Universitaire d’Hématologie, Paris, France

**Keywords:** cytokines, FAS-L, RAE-1, NK-receptors, iNKT

## Abstract

Natural killer group 2D (NKG2D) is a well-characterized activating receptor expressed on many immune cells, including invariant natural killer T (iNKT) cells. These cells were shown to be responsible of liver injury in the model of concanavalin A (Con A)-induced hepatitis, considered to be an experimental model of human autoimmune hepatitis. In this study, we investigated whether NKG2D plays a role in the hepatitis induced by iNKT cell-mediated immune response to Con A. By using killer cell lectin-like receptor subfamily K, member 1 deficient (*Klrk1*^−/−^) mice, we found that the absence of NKG2D reduced the hepatic injury upon Con A administration. This was not due to an intrinsic functional defect of NKG2D-deficient iNKT cells as mice missing NKG2D have normal distribution and function of iNKT cells. Furthermore, increased resistance to Con A-induced hepatitis was confirmed using neutralizing anti-NKG2D antibodies. The reduced pathogenic effect of Con A in the absence of NKG2D correlates with a reduction in pathogenic cytokine production and FAS-Ligand (FAS-L) expression by iNKT cells. We also found that Con A administration led to an increase in the retinoic acid early inducible (RAE-1) surface expression on wild-type hepatocytes. Finally, we found that Con A has no direct action on FAS-L expression or cytokine production by iNKT cells and thus propose that NKG2D-L expression on stressed hepatocytes promote cytotoxic activity of iNKT cells *via* its interaction with NKG2D contributing to hepatic injury. In conclusion, our results highlight NKG2D as an essential receptor required for the activation of iNKT cells in Con A-induced hepatitis and indicate that it represents a potential drug target for prevention of autoimmune hepatitis.

## Introduction

Liver diseases, including autoimmune hepatitis, viral hepatitis, alcoholic liver disease, and primary biliary cirrhosis, afflict >10% of the world population; however, the immunopathogenesis remains largely undefined which limits the efficacy of clinical treatments of these diseases. Concanavalin A (Con A)-induced hepatitis is a well-established experimental murine model (1), which rapidly induces severe immune-mediated hepatitis due to the specific activation of invariant natural killer (iNKT) cells (2).

Invariant natural killer T cell functional subsets are determined by their cytokine profile and their transcriptional programs. Thus, “Th-1 like,” “Th2-like,” and “Th-17 like” iNKT cell subsets have been defined (3). The “Th-1 like” iNKT cells expressing the transcription factor T-bet produce large amounts of interferon (IFN)-γ [and interleukin (IL)-4 at lower levels] and form the majority of hepatic and splenic iNKT cells. “Th2-like” iNKT cells are enriched in the lungs and produce IL-4, IL-9, IL-10, and IL-13. “Th-17-like” iNKT cells produce IL-17, IL-21, and IL-22, are present in the lymph nodes and skin, and require the transcription factor retinoic acid receptor-related orphan receptor-γt (ROR-γt) for their development.

All iNKT cell subset, are positively selected by the MHC-I-like molecule CD1d (4), as indicated by complete absence of iNKT cells in CD1d-deficient mice (5). They express TCRs that consist of an invariant Vα14-Jα18 TCRαβ chain (human Vα24Jα18) paired with a limited number of TCRβ chains, Vβ8, Vβ7, orVβ2 (Vβ11 in humans). The TCRs expressed on mature iNKT cells recognize CD1d-presented glycolipids such as α-galactosylceramide (α-GalCer), a potent activator of both mouse and human iNKT cells (6). The acquisition of the phenotype and function of cells is regulated through three developmental stage defined by the progressive acquisition of CD44 and NK1.1, starting by stage 1 (CD44^−^NK1.1^−^) to stage 2 (CD44^+^NK1.1^−^), and ultimately to stage 3 (CD44^+^NK1.1^+^) iNKT cells (7). During the final stage of iNKT development, cells rapidly express NK lineage receptors such as NK1.1, NK group 2 member D (NKG2D), 2B4, CD94/NKG2A and Ly49 receptors (5). They also acquire NK function as stage 3 NK1.1^+^ iNKT cells produce large amounts of cytokines such as IFN-γ, IL-4, TNF-α (8), allowing them once activated to initiate effector functions and modulate the immune response of other immune cells in microbial infections, autoimmune, allergic diseases, and cancer (9, 10). The iNKT cell subset involved in Con A-induced hepatitis are mature iNKT1 cells (mainly stage 3 iNKT cells) because the type of cytokine they produce where shown to be pathogenic in this model (11–13) and because of their preferential accumulation in the liver where they constitute up to 30% of all T cells and 90% of iNKT cells (5).

In the Con A-induced hepatitis model, in addition to cytokine production (IFN-γ, IL-4, TNF-α), it has been also shown that activated iNKT cells also upregulate FAS-L on their surface and induce hepatocyte apoptosis through the FAS/FAS-L pathway which appears to be an important mechanism for liver damage, as iNKT cells from FAS-mutant *gld*/*gld* mice fail to induce hepatitis (2). However, the mechanisms leading to the induction of FAS-L on the surface of iNKT are partly known (13).

NK group 2 member D is a type II transmembrane-anchored glycoprotein, which has been shown to be an activating or costimulatory receptor expressed on many immune cells such as NK cells, activated CD8 T lymphocytes, and iNKT cells (14–16). In mice, NKG2D-ligands include the retinoic acid early-inducible 1 family of proteins [retinoic acid early inducible 1 (RAE-1)], H60, and MULT1 (17–19). The ligands of NKG2D are known to be “stress-inducible” molecules, induced by cellular transformation, viral infection (20), and/or DNA damage (21). Furthermore, NKG2D serves a fundamental role in the surveillance against microbial infection and cancer (22), but an abnormal activation could also be deleterious by causing autoimmune responses. Indeed, the involvement of NKG2D and its ligands has been revealed in many autoimmune diseases, such as rheumatoid arthritis, celiac disease, and autoimmune diabetes (23–25). The physiological role of NKG2D expressed on the invariant Vα14 iNKT cells in hepatitis is yet to be determined.

In this study, we found that the absence of NKG2D reduced disease severity upon Con A administration that is not due to an altered iNKT cell development in these mice. The contribution of NKG2D in the disease severity is mediated by its interaction with NKG2D-ligands expressed on hepatocytes leading to increased cytokine production and FAS-L expression in iNKT cells and increased cytotoxic potential. Overall, our results indicate that NKG2D promotes the effector function of iNKT cells in this model of liver disease and thus represent a potential drug target for prevention of autoimmune hepatitis.

## Materials and Methods

### Mice

NK group 2 member D-deficient mice on the B6 genetic background have been described elsewhere (26) and were purchased from The Jackson Laboratory. A scheme of heterozygote breeding pairs was chosen to generate littermates of mice of all three *Klrk1* genotypes (+/+, +/−, and −/−). *CD1D*^−/−^ or *Tcra-J*^*tmITg*^ (referred as CD1d^−/−^ and Jα18^−/−^ mice, respectively) on B6 background have been described elsewhere (27, 28). All mice were bred and maintained under specific pathogen-free conditions at our animal facility in compliance with institutional guidelines. Experimental studies were performed in accordance with the Institutional Animal Care and Use Guidelines. The study was approved by the ethics committee “Comité d’Ethique Paris-Nord; C2EA-121,” affiliated to the “Comité National de réflexion Ethique en Expérimentation Animale (CNREEA) et au Ministère de l’Enseignement Supérieur et de la Recherche.”

### Monoclonal Antibodies (mAbs) and Flow Cytometry

Anti-B220 (clone: RA3-6B2) BV510-, PE-, and FITC-conjugated; anti-TCRβ (H57-597) FITC and AF-700 conjugated, anti-Ly49F (HBF-719) PE-conjugated, anti-HSA (M1/69) BV510- and FITC-conjugated, anti-Ly49G2 (4D11) FITC-conjugated, anti-Ly49C/I (5E6) FITC-conjugated, anti-Ly49A (A1) V450-conjugated, and anti-Ly49H (3D10) PECF594-conjugated mAbs were purchased from BD Biosciences. Anti-CD8 (53-6.7) FITC-conjugated, anti-CD62L (MEL-14) PE-conjugated, anti-NKG2A (16A11) APC-conjugated, anti-Ly49D (4E5) FITC-conjugated, anti-RAE-1d (d1.23) PE-conjugated, anti-IL-4 (11B11) BV421-conjugated, anti-IFN-γ (XMG1.2) FITC-conjugated, and anti-TNF-α (MP6-XT22) BV510-conjugated mAbs were purchased from Biolegend. Anti-PD-1 (J43) FITC-conjugated, anti-CD122 (5H4) PE-conjugated, anti-CD4 (RM4-5) PerCP Cy5.5-, PECy7- and PECy5-conjugated; anti-NK1.1 (PK136) PECy7-, PerCPCy5.5- and APC- conjugated; anti-CD69 (H1.2F3) FITC-conjugated, anti-2B4 (eBio244F4) PECy7-conjugated, and anti-CD94 (c18d3) efluor450-conjugated mAbs were purchased from eBioscience. CD1d-α-GalCer tetramers (tet) were produced with streptavidin-APC or -BV421 (BD Biosciences) and used for staining as described previously (29). For intracellular staining, cells were fixed with PBS-2% parafomaldehyde solution, washed, and then permeabilized with PBS-0.2% saponin solution (all from Sigma-Aldrich). Flow cytometry was performed with BD Fortessa instrument and FACSDiva v6.1.2 software (BD Biosciences).

### Mononuclear Cell Preparation

Thymus, pooled PLNs (comprising axillary, sub-axillary, maxillary, inguinal, and popliteal lymph nodes) and spleen were isolated, mechanically disrupted, and filtered through a 40-µm stainless steel mesh to obtain single-cell suspensions. Livers were dissected (gall bladder removed) and passed through a 40-μm-pore cell strainer. Cells were washed twice and suspended in PBS-FCS 5%, overlaid in Lymphocyte Separation Medium (Eurobio) and centrifuged at 2,000 rpm for 25 min at room temperature. Hepatic mononuclear cells were collected from the interface and washed.

### RNA Extraction

Each liver tissue was disrupted on ice with a tissueRuptor (Qiagen) in buffer RLT added with β-mercaptoethanol. Total mRNA was extracted and purified using RNeasy Fibrous Tissue mini kit (Qiagen), according to the manufacturer’s instructions.

### *In Vivo* iNKT Cell Stimulation With Free α-GalCer

α-Galactosylceramide (KRN 7000) was a gift from the KIRIN company and was dissolved in PBS at a concentration of 220 µg/ml. Twelve-week-old mice were injected i.p. with 0.01, 0.02, 0.04, or 0.2 µg of α-GalCer in a final volume of 200 µl of PBS or vehicle as controls. After 2 h, splenocytes and hepatic mononuclear cells were prepared and incubated for 2 h with Brefeldin A (Sigma-Aldrich) at 5 µg/ml for intracellular staining.

### *In Vitro* Activation of iNKT Cells

For measurements of intracellular cytokines, cells were stimulated with 50 ng/ml phorbol-12-myristate-13-acetate (Sigma-Aldrich), 1 µM ionomycin (Cell Signaling Technologies), or 10 µg/ml of Con A (30), in the presence of 5 µg/ml brefeldin A (Sigma-Aldrich) for 4 h. For measurement of cytokine released in the supernatant, we performed ELISA as described previously (31).

### Induction of Con A-Induced Hepatitis

Concanavalin A (Sigma) was dissolved in pyrogen-free PBS and i.v. injected to mice through the tail vain at a dose of 15 or 25 mg/kg corresponding to no lethal and lethal dose, respectively (2). Sera from individual mice were obtained from 2 to 24 h after Con A injection. Serum alanine aminotransferase (ALT) activity was determined using “Sigma ALT-detection kit” according to manufacturer’s protocol.

### Histological Examination and Fluorescence Microscopy

For histological examination, hematoxylin/eosin staining of paraffin-embedded liver sections was performed as described previously (2). RAE-1 was detected in immersion fixed frozen sections of mouse liver using Goat Anti-Mouse RAE-1 Pan Specific Antigen Affinity purified Polyclonal Antibody (Clone AF1136) at 5 µg/ml overnight at 4°C. DAB Cell & Tissue Staining Kit (brown; Catalog # CTS008) was used and counterstained with hematoxylin (blue).

For fluorescence microscopy, liver cells suspensions from mouse non-treaded or treated with Con A were plated on poly (l-lysine)-coated coverslips (Sigma-Aldrich). Cells were then fixed with 4% paraformaldehyde for 30 min and permeabilized with 0.1% SDS or Triton X-100 for 10 min, followed by blocking with 10% FBS for 20 min. The fixed cells were stained with anti-mouse RAE-1 Pan Specific Alexa Fluor 647-conjugated mAb (Clone #186107), or Rat IgG2A Alexa Fluor 647-conjugared mAbs as isotype control (Clone # 54447), diluted in PBS containing 1 mg/ml BSA. Nuclei were stained with DAPI (Molecular Probes, Invitrogen). Coverslips were mounted with Vectashield (Vector laboratories) and analyzed with a fluorescence microscope (Carl Zeiss LSM-510) and LSM Image Examiner software (Carl Zeiss).

### Statistics

Statistical analyses were performed using PRISM 6 software (GraphPad Software, Inc., CA, USA). Results are expressed as the means ± SD. The statistical significance of differences between experimental groups was calculated by was assessed by the non-parametric Mann–Whitney *U* or the test a one-way ANOVA with a Tukey’s post test. *P* values correspond to the following annotation: *P ≤ 0.05, **P ≤ 0.01, and ***P ≤ 0.001.

## Results

### Normal Development of iNKT Cells in the Absence of NKG2D

**Figure 1:**
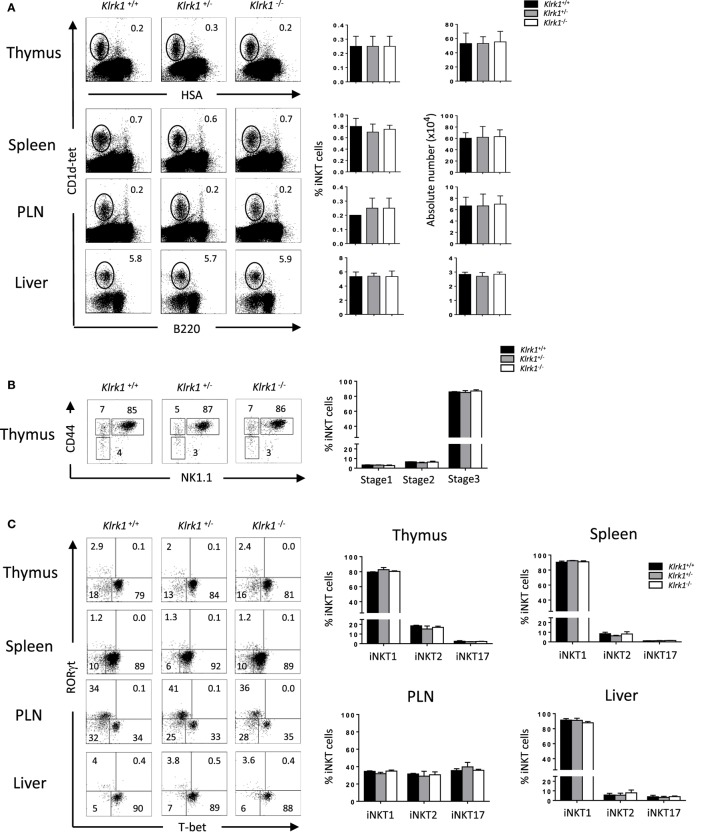
Normal development of invariant natural killer T cell (iNKT) cells in the absence of NK group 2 member D **(A)**. Representative Flow cytometry analysis of iNKT cells in the thymus, spleen, PLN, and liver. iNKT cells are defined as tet^+^HSA^low^ in the thymus or tet^+^B220^−^ in the periphery. Histograms show frequency of iNKT cells. **(B)** Representative dot plot of NK1.1/CD44 gated on thymic tet^+^HSA^low^ cells. Histogram plots show the frequency of iNKT cells at stage 1 (CD44^−^NK1.1^−^), stage 2 (CD44^+^NK1.1^−^), and stage 3 (CD44^+^NK1.1^+^) of iNKT cell development. **(C)** Representative staining of iNKT1 (T-bet^+^), iNKT2 (RORγt^−^/T-bet^−^), and iNKT17 (RORγt^+^) subsets among iNKT cells at the indicated organs. Frequencies of these different subsets are represented in histograms. Data are from five experiments where three mice aged 5–6 week were used per experiment and presented as mean ± SD. Numbers represent percentages.

To study the role of NKG2D in Con A-induced hepatitis, we used *Klrk1*^−/−^ mice deficient in NKG2D protein described in a previous study (26). We first assessed the frequency and numbers of iNKT cells determined by using CD1d-tetramers that allow identifying iNKT cells based on their TCR specificity (29). We found in the thymus, spleen, PLNs, and liver, comparable frequencies and absolute numbers of iNKT cells in *Klrk1*^−/−^, *Klrk1*^+/+^, and *Klrk1*^+/−^ littermate controls (Figure 1A). Frequencies of CD4^+^ and CD4^−^CD8^−^ double negative (DN) iNKT cell subsets in *Klrk1*^−/−^ mice were not different from those of *Klrk1*^+/+^ and *Klrk1*^+/−^ littermate controls in all organ tested.

It has been shown that T cell-surface costimulatory molecules, such as CD28 and ICOS, play a role in iNKT cell development through the induction of T-bet (32). We next sought to assess if the absence of NKG2D, a costimulatory activating receptor, could impair the maturation of iNKT cells. We found comparable frequencies and absolute numbers of early precursor stage 1, stage 2, and stage 3 iNKT cells, the latter being the fully mature and the most abundant iNKT cells in the adult thymus, in *Klrk1*^−/−^, and *Klrk1*^+/−^, compared to *Klrk1*^+/+^ mice (Figure 1B, and data not shown).

Because NKG2D is expressed on the majority of iNKT1 cells, we hypothesized that it might play a role in their terminal maturation or maintenance. We, thus, used T-bet and RORγt expression to evaluate the composition of iNKT subsets in the absence of NKG2D. As shown in Figure 1C, iNKT1 (T-bet^+^), iNKT17 (RORγt^+^), and iNKT2 (T-bet^−^RORγt^−^) iNKT cell frequencies and numbers are similar in the thymus, spleen, PLNs, and liver of *Klrk1*^−/−^ mice compared to *Klrk1*^+/−^ or *Klrk1*^+/+^ littermate control.

We also assessed proportions and numbers of other effector cells that express NKG2D, such as NK and CD8^+^ cells in the thymus and spleen and found that the proportions and absolute numbers of CD8^+^ and NK cells remain unchanged in NKG2D-deficient mice compared to wild-type mice (Figure S1 in Supplementary Material) confirming previous results (26).

Taken together, these results show that NKG2D is dispensable for the early developmental stages and terminal maturation of thymic and peripheral iNKT cells. They also indicate that the generation all iNKT cells subsets, the one expressing (iNKT1) or non-expressing (iNKT2 and iNKT17) NKG2D, is not altered in the absence of NKG2D.

### The Expression of iNKT Cell Phenotypic Makers Is Preserved in the Absence of NKG2D

**Figure 2:**
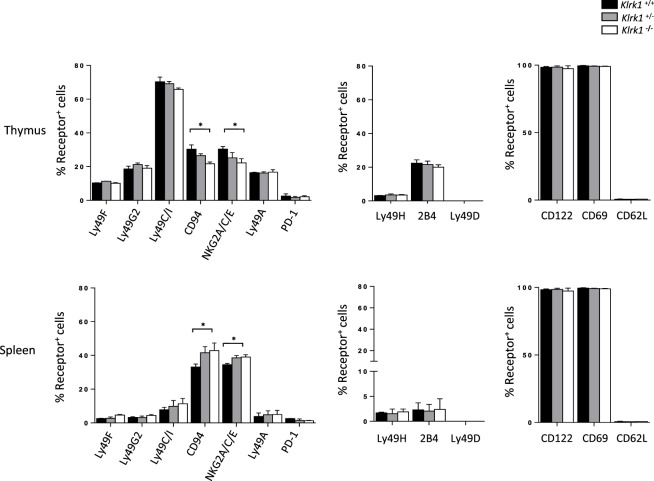
The expression of invariant natural killer T cell (iNKT) cell phenotypic makers is preserved in the absence of NK group 2 member D. Frequencies of inhibitory (left) and activating (middle) NK-receptors, and other activating receptors (right) among NK1.1^+^ iNKT cells in the thymus and spleen. iNKT cells are defined as tet^+^HSA^low^ in the thymus or tet^+^B220^−^ in the spleen. Data are from five experiments where three mice aged 5- to 6-week old were used per experiment and presented as mean ± SD. Numbers represent percentages. Significance is represented by an asterisk and was evaluated with non-parametric Mann–Whitney *U* test.

To address the relevance of NKG2D on shaping the NK-cell receptor repertoire on iNKT cells, we analyzed the expression of inhibitory and activating receptor on NK1.1^+^ iNKT cells in the absence of NKG2D expression. Among the inhibitory receptor tested we observe normal (Ly49F, Ly49G2, and Ly49A) or minor (CD94/NKG2A/C/E) differences of the expression of these receptors in iNKT cells from *Klrk1*^−/−^ mice (Figure 2, and Figure S2A in Supplementary Material) compared to *Klrk1*^+/+^ mice. The minor difference for CD94/NKG2A/C/E expression is organ specific because, while slightly upregulated in the thymus, the opposite effect is observed in the spleen (Figure 2; Figure S2A in Supplementary Material) and liver (Figure S2B in Supplementary Material). We also found that basal expression of the inhibitory costimulatory receptor PD-1 was not altered in NKG2D-deficient thymic and splenic iNKT cells (Figure 2; Figure S2A in Supplementary Material).

On the other hand, frequencies of iNKT cells expressing activating receptors tested (Ly49H, 2B4, and Ly49D) were similar in the spleen and thymus of all three genotypes (Figure 2; Figure S2C in Supplementary Material, upper panel). We also found that the frequency of iNKT cells expressing memory T cell (CD44^high^CD62L^low^) and activation (CD122, CD69) markers was similar between genotypes in both the thymus and spleen (Figure 2; Figure S2C in Supplementary Material, lower panel).

Taken together, these data indicate that NKG2D expression is dispensable for phenotypic maturation of iNKT cells.

### NK1.1^+^ iNKT Cells Generated in the Absence of NKG2D Keep Their Potential to Produce Cytokine Upon Stimulation

**Figure 3:**
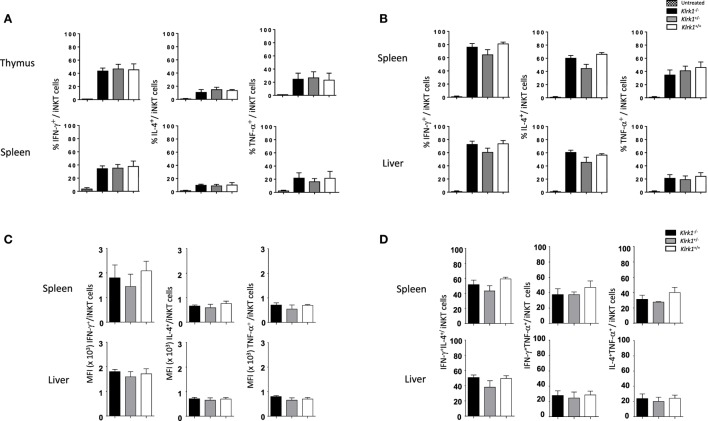
Unchanged cytokine production capabilities of invariant natural killer T cell (iNKT) cells in the absence of NK group 2 member D. **(A)** Frequencies of interferon (IFN)-γ, interleukin (IL)-4, and TNF-α production by iNKT cells *in vitro*, assessed by intracellular staining, in the thymus and spleen upon PMA/ionomycin stimulation for 4 h. **(B)** Frequencies of IFN-γ, IL-4, and TNF-α production by iNKT cells, assessed by intracellular staining, in the spleen and liver *in vivo* 2 h after i.p. injection of α-galactosylceramide. **(C)** Mean florescence intensity (MFI) of IFN-γ, IL-4, and TNF-α expression by iNKT cells from the experiment in **(B)**. **(D)** Frequencies of IFN-γ^+^IL-4^+^, IFN-γ^+^TNF-α^+^, and IL-4^+^TNF-α^+^ producing iNKT cells from the experiment in **(B)**. iNKT cells are defined as tet^+^HSA^low^ in the thymus or tet^+^B220^−^ in the spleen or liver. Data are from three experiments where three mice aged 5- to 6-week old were used per experiment and presented as mean ± SD.

To test if the function of iNKT cells is altered in the absence of NKG2D, we first assessed their cytokine production *in vitro* after PMA/ionomycin stimulation. As show in the histograms of Figure 3A and in representative dot plots in Figure 3A in Supplementary Material, the frequencies of IFN-γ-, TNF-α-, and IL-4-positive cells among NK1.1^+^ iNKT cells from *Klrk1*^−/−^, *Klrk1*^+/−^, and *Klrk1*^+/+^ littermate controls were not different in both organs of each genotype.

We next sought to assess the consequences of NKG2D deficiency on iNKT cells function *in vivo* with the rationale that NKG2D activation on iNKT cells can act synergistically with IL-12 to promote a Th-1-skewed response (33). To do so, injected intraperitoneally optimal dose of α-GalCer (0.2 µg as determined in Figure S3B in Supplementary Material) and found that frequencies of cytokine positive iNKT cells were not statistically different in the spleen and liver of α-GalCer-treated or vehicle-treated *Klrk1*^−/−^, *Klrk1*^+/−^, and *Klrk1*^+/+^ littermate controls (Figure 3B; Figure S3C in Supplementary Material). Because a strong TCR stimulation through optimal dose of α-GalCer might have masked a possible contribution of NKG2D, we used a suboptimal dose of α-GalCer (0.02 µg) and found that cytokine production by iNKT cells is also not altered in NKG2D-deficient iNKT cells (data not shown).

We also measured mean fluorescence intensity (MFI) of cytokine expressed by iNKT, assessed by intracellular staining, upon α-GalCer injection and found that MFI of IFN-γ, IL-4, and TNF-α are comparable in NKG2D-deficient mice and wild-type mice (Figure 3C). We also compared the frequencies of iNKT cells producing IFN-γ^+^IL-4^+^, IFNγ^+^TNF-α^+^, and IL-4^+^TNF-α^+^ in wild-type and NKG2D-deficient mice and found similar distribution (Figure 3D). These results indicate that NKG2D-deficient iNKT cells do not differ in their capabilities to produce cytokine on a per cell basis and do not lose their polyfunctionality.

Altogether, these *in vitro* and *in vivo* results show that iNKT cells that develop in the absence of NKG2D keep their potential to produce cytokines.

### Reduced Hepatic Injury and Increased Survival in the Absence of NKG2D-Mediated Signals in Con A-Induced Hepatitis

**Figure 4:**
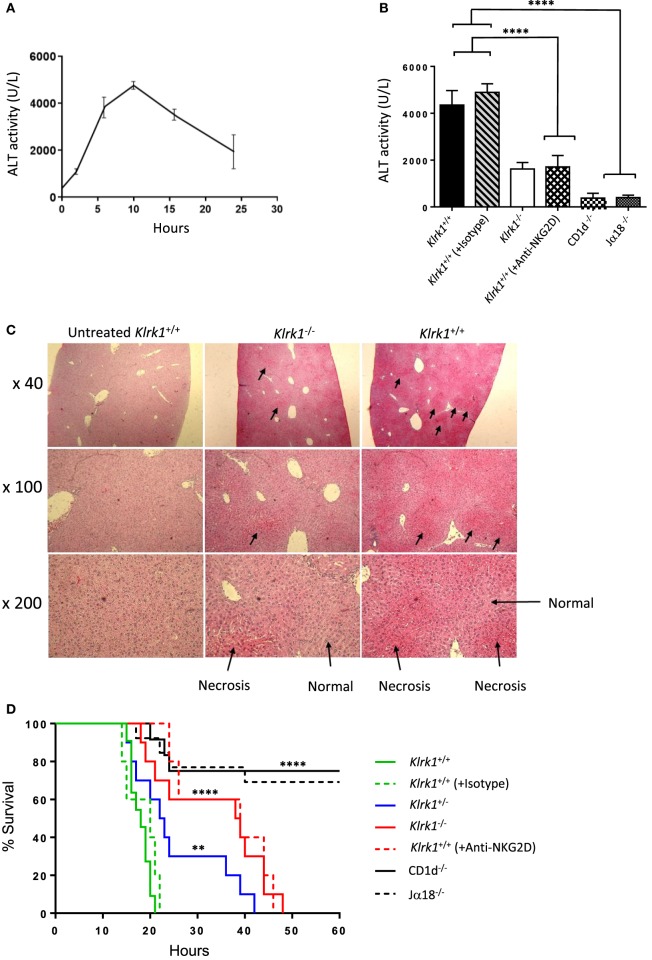
Reduced hepatic injury and increased survival in the absence of NK group 2 member D (NKG2D)-mediated signals in concanavalin A (Con A)-induced hepatitis **(A)**. Kinetics of serum transaminase (ALT) levels in B6 mice after Con A administration (15 mg/kg). **(B)** ALT levels 10 h after Con A administration (15 mg/kg) in mutant, wild-type, and wild-type mice injected or not with anti-NKG2D neutralizing antibodies. **(C)** Light micrographs of the liver 10 h after administration of Con A (15 mg/kg) with hematoxylin and eosin stating (40×, 100×, 200×) are shown. **(D)** Survival curve of mice injected with lethal dose (25 mg/kg) of Con A are shown. Results are from four experiments where five mice of each genotype were used per experiment. Significance compared to wild type is represented by asterisks and was evaluated by a one-way ANOVA with a Tukey’s post test.

Several experimental results demonstrated that iNKT cells are key effector cells in Con A-mediated liver damage (2, 13). To explore the possible contribution of NKG2D expressed on iNKT cells to the pathogenesis of Con A-induced hepatitis, we i.v. injected non-lethal doses (15 mg/kg) of Con A into NKG2D-deficient mice and measured serum ALT levels after Con A injection. In preliminary experiments, elevated serum levels of ALT were first observed 4–5 h after injection and peaked at 8–12 h after injection (Figure 4A). We thus measured serum ALT levels 10 h after Con A injection and as shown in Figure 4B, serum ALT levels were markedly reduced in *Klrk1*^−/−^ as compared to *Klrk1*^+/+^ mice. CD1d^−/−^ or Jα18^−/−^ mice, both missing iNKT cells and resistant to Con A-induced hepatitis, were used as controls. Histological findings of degenerative changes in the liver were clearly correlated with the serum ALT levels (Figure 4C). In fact, histological examination showed focal and mild injury in *Klrk1*^−/−^ mice whereas in *Klrk1*^+/+^ mice a diffuse and massive degenerative change was observed as previously reported (2). We next evaluated whether reduced liver injury in *Klrk1*^−/−^ mice would have a positive impact on mice survival after injection of lethal doses of Con A (30 mg/kg). As shown in Figure 4D, whereas all wild-type mice died between 15 to 20 h, 20% of *Klrk1*^−/−^ mice died within this time frame, and 60% of them were still alive at 40 h, clearly demonstrating the less sensitivity of *Klrk1*^−/−^ mice to Con A-induced hepatitis. The pretreatment of *Klrk1*^+/+^ mice with neutralizing anti-NKG2D antibodies reduced ALT levels (Figure 4B) and increased the survival rates to the same level as in genetically modified *Klrk1*^−/−^ mice (Figure 4D), indicating that this beneficial effect observed in *Klrk1*^−/−^ mice could not be attributed to a developmentally related alteration in the function of iNKT cells.

Overall, these results indicated that NKG2D expressed on iNKT cells contribute substantially to the Con A-induced hepatitis.

### Reduction of Cytokine Production and Fas-L Expression in the Absence of NKG2D in ConA-Induced Hepatitis

**Figure 5:**
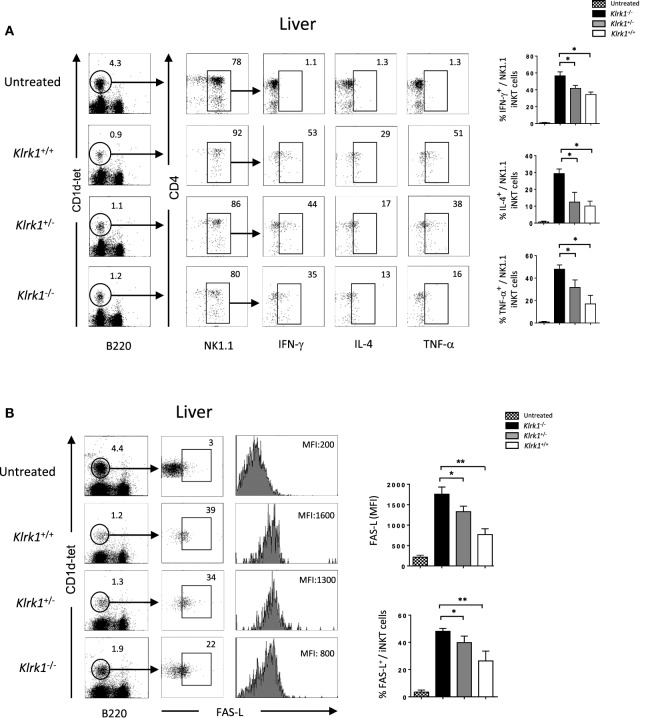
Reduction of cytokine production and FAS-L expression by liver invariant natural killer T cell (iNKT) cells in the absence of NK group 2 member D upon concanavalin A (Con A) administration, Representative intracellular staining of interferon (IFN)-γ, interleukin (IL)-4, and TNF-α production **(A)**, or FAS-L expression **(B)** by liver NK1.1^+^ iNKT after 2 h of i.v. administration of Con A (15 mg/kg). iNKT cells are defined as tet^+^B220^−^. The frequencies of positive cells and mean florescence intensity (MFI) of FAS-L are shown in the histograms. Results are from three experiments where three mice of each genotype were used per experiment and presented as mean ± SD. Numbers represent percentages. Significance is represented by an asterisk and was evaluated with non-parametric Mann–Whitney *U* test.

Different cytokines, such as TNF-α, IFN-γ and IL-4, that are released upon application of Con A have been suggested to mediate hepatic inflammation and parenchymal necrosis (11–13). We examined intracellular cytokine production by liver iNKT cells 2 h after injection of non-lethal doses of Con A. We found that the frequency of IFN-γ producing liver NK1.1^+^ NKT cells from *Klrk1*^−/−^ mice dropped to 35±4% SD compared to 50±4% SD observed in wild-type mice (Figure 5A). A more drastic drop for NK1.1^+^ NKT cells producing TNF-α was observed as only 16±4% SD of cells from *Klrk1*^−/−^ mice produced TNF-α compared to 50±4% SD observed in wild-type mice. A reduction in IL-4 cytokine production was also observed and the frequency of NK1.1^+^ iNKT cells producing IL-4 dropped from 30±4% SD to 10±4% SD in cells from *Klrk1*^+/+^ versus *Klrk1*^−/−^, respectively. Such fluctuation in cytokine production was not observed in spleen iNKT cells (Figure S4A in Supplementary Material).

Different studies have shown that FAS/FAS-L-mediated cytotoxicity by iNKT cells is critically important for hepatic injury in the Con A model (2, 13). As shown in Figure 5B, hepatic iNKT cells rapidly upregulate cell-surface FAS-L expression upon Con A administration. In the absence of NKG2D in *Klrk1*^−/−^ mice, this upregulation is three time lower than the one observed in *Klrk1*^+/+^ mice (MFI of 1,800 ± SD vs 600 ± SD in iNKT cells from *Klrk1*^+/+^ and *Klrk1*^−/−^ mice, respectively). Such upregulation was not observed in spleen iNKT cells (Figure S4B in Supplementary Material), although they produced cytokine in response to Con A (Figure S4A in Supplementary Material).

Overall, our results indicate that NKG2D deficiency reduced cytokine production and FAS-L expression by liver iNKT cells upon Con A injection. Because these produced cytokines and FAS-L are key mediators of the disease, these findings are likely to explain the reduced liver injury and increased survival we observed in *Klrk1*^−/−^ mice.

### Crosstalk Between iNKT Cells and Hepatocytes Occur *via* NKG2D–NKG2D-L Interaction in Con A-Induced Hepatitis

**Figure 6:**
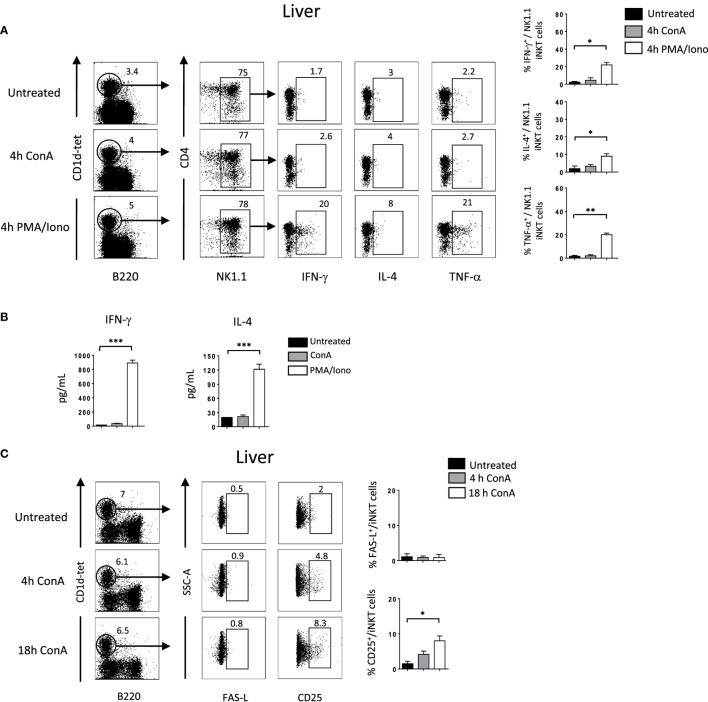
Cytokine production and FAS-L expression by liver invariant natural killer T cell (iNKT) cells is not induced directly *in vitro* by Concanavalin A (Con A) **(A)**. Representative intracellular staining of interferon (IFN)-γ, interleukin (IL)-4, and TNF-α production expression by liver NK1.1^+^ iNKT after 4 h incubation in the presence of Con A (10 µg/ml) or PMA/ionomycin. iNKT cells are defined as tet^+^B220^−^. The frequencies of positive cells are shown in the histograms. **(B)** Cytokine level in the supernatant by liver mononuclear cells after O/N incubation with Con A or PMA/ionomycin at the previously indicated concentration. **(C)** Representative cell-surface staining of FAS-L expression by liver NK1.1^+^ iNKT after 4 or 18 h incubation in the presence of Con A (10 µg/ml). Results are from three to four experiments where three mice of each genotype were used per experiment and presented as mean ± SD. Significance is represented by an asterisk and was evaluated with non-parametric Mann–Whitney *U* test.

**Figure 7:**
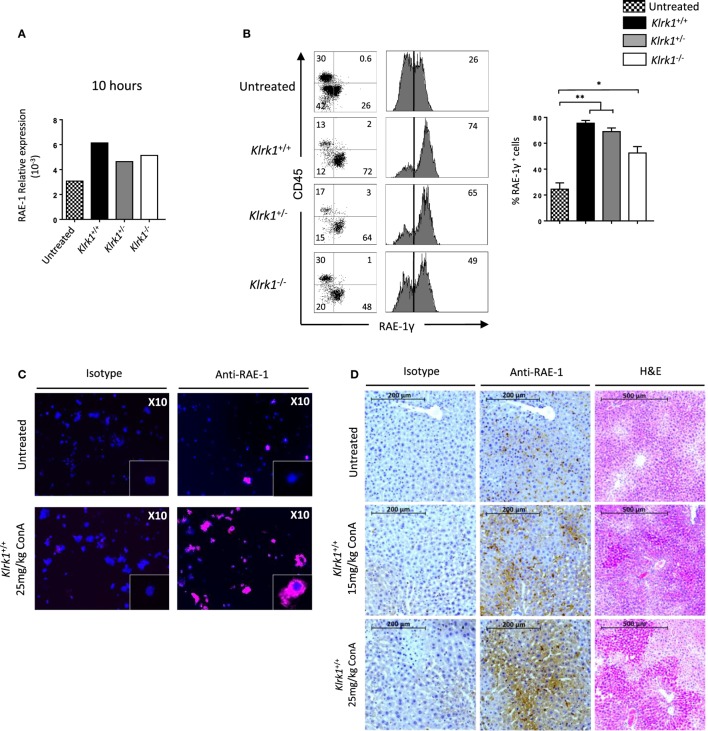
Retinoic acid early inducible 1 (RAE-1) expression is upregulated upon concanavalin A (Con A) administration *in vivo* **(A)**. Relative expression of RAE-1 transcript in liver tissue in non-injected, or 10 h after Con A administration (25 mg/kg). **(B)** Representative histogram staining of RAE-1 expression at the cell-surface of hepatocytes in non-injected or after 8 h after Con A administration (25 mg/kg). **(C)** RAE-1 expression assessed in hepatocytes by fluorescent microscopy in non-injected or 3 h after Con A administration (25 mg/kg). **(D)** Histological examination of RAE-1 expression on hepatic sections from mice non-injected or 8 h after Con A administration (15 or 25 mg/kg). Note that increased doses of Con A correlate with increased RAE-1 expression and liver degenerative changes as assessed by H&E staining. Results are from four experiments where three mice of the indicated genotype were used per experiment and presented as mean ± SD. Significance is represented by an asterisk and was evaluated with non-parametric Mann–Whitney *U* test.

So far our results indicate that NKG2D is important to induce cytokine production and FAS-L expression by iNKT cells upon Con A injection. To assess if Con A could induce directly these biological changes in iNKT cells, we incubated liver mononuclear cells in the presence of Con A and measured cytokine production and FAS-L expression. Four hours after Con A incubation, we found that NK1.1^+^ iNKT cells produce lower amount of cytokines compared to what observed after PMA/ionomycin stimulation (around five times less IFN-γ and IL-4, and 10 times less TNF-α), indicating the inefficacy of Con A to stimulate iNKT cells (Figure 6A). Our results are confirmed by measuring cytokine production in the supernatant by ELISA as we barely detect cytokine produced in the presence of Con A compared to PMA/ionomycin (Figure 6B). We were also not able to detect FAS-L expression on the surface of iNKT cells after 4 or 18 h incubation in the presence of Con A, while we observed CD25 expression indicating activation of these cells (Figure 6C). The limited cytokine production and absence of FAS-L expression are also observed in spleen iNKT cells (Figure S5 in Supplementary Material). These *in vitro* results suggest strongly that Con A could not promote directly the pathogenicity of iNKT cells *in vivo* upon Con A injection. We thus reasoned that NKG2D action will be mediated by its interaction with NKG2D-ligands expressed on stressed hepatocytes. We thus investigated the expression of RAE-1, one of the major ligand of NKG2D, in liver cells. We found that RAE-1 transcripts are upregulated upon Con A administration (Figure 7A). At the protein level, we found by FACS staining that RAE-1 is expressed at basal level on hepatocytes and that its expression is upregulated upon Con A injection (Figure 7B). By florescence microscopy, we were able to confirm these results by visualizing RAE-1-expressing hepatic cells upon Con A administration compared to basal level in non-treated mice (Figure 7C). Finally, histological examination *in situ* showed a dose-dependent increase in RAE-1 expression on hepatocyte section upon administration of a non-lethal and lethal dose of Con A (Figure 7D). The higher expression of RAE-1 observed at lethal dose of Con A correlate with the higher degenerative changes observed by H&E staining.

Overall, these results confirm the hypothesis that the cytotoxic activity of iNKT mediated by NKG2D upon Con A administration is promoted by its interaction with NKG2D-L expression on stressed hepatocytes and contribute to hepatic injury in this murine model.

## Discussion

Invariant natural killer T cell cells preferentially distribute to the liver and have been shown to trigger hepatic injury following Con A injection (2). To elucidate the role of NKG2D in this model, we used deficient mice for this receptor and report that liver injury was reduced significantly in the absence of the NKG2D, correlating with increased survival. Our results show that NKG2D plays a role in the disease development by augmenting the cytotoxic potential of iNKT cells through induction of FAS-L on their surface after Con A injection. In addition, we found that NKG2D is also necessary to upregulate the production of inflammatory cytokines, such as IFN-γ, IL-4, and TNF-α by iNKT cells. Mechanistically, we show that activation of iNKT cells by Con A involves the upregulation NKG2D-L on hepatocyte and their interaction with NKG2D on iNKT cells.

The ontogenetic signals that lead to the development and functional changes of iNKT cells are poorly understood. Even though mature thymic, splenic, and hepatic iNKT1 cells are phenotypically similar, they express NK-cell receptors at very different levels, besides behaving as different functional populations. These differences might be due to distinct agonistic ligands that provide different signals depending on the anatomical location and microenvironment. There is evidence that NKG2D-ligands can be expressed to a limited extent on healthy cells in the thymus, liver, spleen, lungs, intestine, and other organs of mice and humans (34, 35). We could, therefore, hypothesize that NKG2D/NKG2D-ligand interactions in the thymus and peripheral tissues might deliver signals to iNKT cells that would exert an influence on their development and functional differentiation. Our study explored the role of NKG2D in the development and function of iNKT cells in the absence of NKG2D by using *Klrk1*^−/−^ mice and show that NKG2D do not affect the maturation or the production of different iNKT cell subsets (iNKT1, iNKT2, and iNKT17) including the one expressing NKG2D (iNKT1). We found, however, that NKG2D slightly shapes the repertoire of NK-cell receptor on iNKT cells, especially of CD94, whose expression was decreased in thymic iNKT cells and, conversely, increased in splenic and liver iNKT cells. These differences could be due to tissue-specific regulation on the acquisition or selection of the NK receptor repertoire. The fact that thymic iNKT cells express NK-receptors at higher frequencies points to this direction. CD94 is a co-receptor that is expressed as a heterodimer along with NKG2A, to form an inhibitory receptor, or with NKG2C or NKG2E, forming an activating receptor. However, there is evidence that thymic, splenic, and hepatic iNKT cells preferentially express NKG2A/CD94 in B6 mice (36, 37). With this regard, we found that the expression of NKG2A parallel the one of CD94 as assessed with anti- NKG2A/C/E antibodies. A previous study provided evidence of a moderate alteration of NK-cell differentiation in NKG2D-deficient mice (26). Another study provided evidence for an important regulatory role of NKG2D in the development of NK cells (38). This latter finding points to different molecular mechanisms of development between NK and iNKT cells. For instance, an explanation could be that NK cells express NKG2D in early stages of development (38), whereas this receptor is not expressed early on common iNKT cell precursors but relatively late during differentiation of iNKT1 cells.

Our findings reveal that iNKT cells that developed in the absence of NKG2D have no intrinsic functional defect. In fact, our results reveal that iNKT cells from *Klrk1*^−/−^ mice keep their Th-1-like profile upon stimulation and are as efficient as iNKT cells from wild-type mice to rapidly produce large amounts of cytokines response *in vitro* in response to PMA/ionomycin and *in vivo* to an optimal dose of a specific antigen. Since 1/NK-receptors can regulate TCR signaling threshold to antigen, 2/NKG2D increases IFNγ production of NK cells stimulated with suboptimal doses of IL-12, 3/the activation of iNKT cells by α-GalCer is IL-12-mediated (9, 39), it is possible that the threshold of activation of iNKT cells could be impaired in NKG2D deficiency. We excluded this possibility as we used suboptimal concentrations of α-GalCer but were not able to shed any difference in the response of iNKT cells in the absence of NKG2D.

Having excluded an impact of NKG2D on iNKT cell development, there is a possibility that some functions of NKG2D-deficient iNKT cells could be compromised in the context of disease. NKG2D is a potent costimulatory receptor of TCR-dependent activation of cytotoxic CD8^+^ T cells, γδ T cells, and NKT cells (22). It has been shown that the expression of NKG2D is required for disease induction in murine models of primary Hepatitis B virus infection (40, 41) and liver inflammation in atherosclerosis (42). In our present study, we used a model of Con A-induced liver injury to assess the role of NKG2D-expressing iNKT cells in a pathologic setting. In this model, iNKT cells are pathogenic through their cytotoxic FAS-L mediated action and their cytokine production that act directly or indirectly on hepatocytes. We found that upon Con A injection in the absence of NKG2D cytokine production by iNKT cells is reduced and FAS-L expression is downregulated (by three) compared to what observed in the presence of NKG2D. The lower response of iNKT cells is accompanied by a reduced liver injury and an increased survival and point out on the role of NKG2D in Con A-induced hepatitis. Our results could not be interpreted as result of a developmental and/or functional defect of iNKT cells in the absence of NKG2D as experiments performed with anti-NKG2D neutralizing antibodies showed a reduced activation of iNKT cells and a resistance of Con A-induced hepatitis similar to the one observed in genetically modified *Klrk1*^−/−^ mice.

Our study provides a mechanism by which NKG2D play a role in Con A-induced hepatitis. The model we propose implicate hepatocytes that are stimulated by Con A and express NKG2D-L including RAE-1 (Figure S6 in Supplementary Material). As indicated by previous studies, engagement of NKG2D with its ligands leads to phosphorylation of the adaptor protein DAP12 (DAP 10 in humans) and recruitment of phoshpoinositide-3-kinase, triggering a downstream signaling with the subsequent cytotoxic response and IFN-γ production (43). For iNKT cells, the cytotoxic response is mediated by FAS-L expression upon NKG2D–NKG2D-L interaction. Hepatic cells are then killed upon FAS-L and FAS interaction, the latter expressed constitutively. IL-4 produced by iNKT cells also contributes to potentiate FAS-L expression (13). IFN-γ and TNF-α could act directly on hepatocyte to cause liver injury (12, 30, 44). The absence of NKG2D in *Klrk1*^−/−^ mice shut off the aforementioned cascade of event reducing FAS-L expression and cytokine production leading to reduced iNKT cell cytotoxic potential and liver injury. The lesser expression of RAE-1 we observed upon Con A injection in *Klrk1*^−/−^ compared to *Klrk1^+/+^* is likely the consequence of a lesser inflammatory environment induced by iNKT cells in the absence of NKG2D expression. In this model, a direct action of Con A on iNKT cells is limited if not absent as assessed by *in vitro* stimulation experiment showing limited amount of cytokine produced and absence of FAS-L upregulation.

Although our results show the involvement of NKG2D in the development of Con A-induced hepatitis, the participation of NKG2D/RAE-1 (and other ligands) appeared to be partial and the survival was not equivalent to that of NKT cell-deficiency. This indicates the existence other potential of cytocidal molecules/mechanisms expressed by NKT cells. With this regard, a recent report showed that TNF superfamily receptor OX40 triggers iNKT cells pyroptosis and liver injury (45). Interestingly, Con A injection failed to induce liver injury and iNKT cell depletion, demonstrating an important role for OX40/NKT cells in liver injury in this model.

It is worth mentioning that the role of iNKT cells and NKG2D in the liver is not unambiguous. A recent study showed that iNKT cells are required to protect the liver from CCL_4_-induced fibrosis treated with IL-30 (46). Reminiscent to our study, iNKT cells require NKG2D surface expression to inhibit liver fibrosis after IL-30 treatment by selectively removing CCL_4_-activated hepatic stellae cells through an NKG2D/RAE-1interaction. On the other hand, a recent study showed that NKG2D, known to have antitumorigenic functions, promotes tumor growth in a model of hepatocellular carcinoma (47). Also, NKG2D promoted natural killer cell-mediated fulminant hepatitis in mice (48). In this polyinosinic:polycytidylic acid/d-galactosamine-induced model of hepatitis, it was shown that simultaneous knockdown of multiple ligands of NKG2D alleviated the disease.

Overall, our result provides the first examination of the role NKG2D in the development and function of NKG2D expressed on iNKT cells. We found no intrinsic defect in the functional capabilities of iNKT cells developed in the absence of NKG2D, even though we observed a minor alteration of the expression of CD94/NKG2A. We also report a critical role for NKG2D in controlling iNKT cell response in a model of Con A-induced hepatitis and provided new insight in the comprehension of the mechanism of action of iNKT cell in this hepatitis model providing novel strategies for clinical applications.

## Author Contributions

DD and VOP designed, performed, and analyzed the experiments; JK designed, performed, analyzed, guided experiments, and addressed editor/referees’ comments; KB designed, analyzed the experiments, wrote the paper, and supervised the study; VP performed experiments; MA and AT analyzed the experiments and provided essential knowledge.

## Conflict of Interest Statement

The authors declare that the research was conducted in the absence of any commercial or financial relationships that could be construed as a potential conflict of interest.

## Supplementary Material

Figure S1 Frequency and numbers of CD8^+^ and NK cells in the absence of NK group 2 member D. Representative Flow cytometry analysis of CD8/TCRβ and NK1.1/TCRβ staining in the thymus and spleen. Histograms show frequencies of CD8^+^ cells (CD8^+^/TCRβ^+^) and NK cells (NK1.1^+^/TCRβ^−^) as defined by the gates. Data are from five experiments where three mice aged 5- to 6-week were used per experiment and presented as mean ± SD. Numbers represent percentages.

Figure S2 Phenotypic characterization of invariant natural killer T cell (iNKT) cells in the absence of NK group 2 member D. **(A)**. Representative staining of inhibitory NK-receptors among NK1.1^+^ iNKT cells in the thymus and spleen. **(B)** Representative staining of CD94 and NKG2A/C/E inhibitory NK-receptors among NK1.1^+^ iNKT cells in the liver. Histograms show frequencies. **(C)** Representative staining of activating NK-receptors (upper panel) and other activating receptors (lower panel) among NK1.1^+^ iNKT cells in the thymus and spleen. Numbers represent percentages. Data are representative of five experiments where three mice aged 5- to 6-week old were used per experiment and presented in histograms as mean ± SD. Significance is represented by an asterisk and was evaluated with non-parametric Mann–Whitney *U* test.

Figure S3 Cytokine production capabilities of invariant natural killer T cell (iNKT) cells in the absence of NK group 2 member D. **(A)** Representative staining of interferon (IFN)-γ, interleukin (IL)-4, and TNF-α production by iNKT cells *in vitro*, assessed by intracellular staining, in the thymus and spleen upon PMA/ionomycin stimulation for 4 h. **(B)** IFN-γ production by spleen iNKT cells assessed by intracellular staining in response to increased doses of α-galactosylceramide (α-GalCer) injected i.p. in normal B6 mice. **(C)** Representative staining of IFN-γ, IL-4, and TNF-α production by iNKT cells in the spleen and liver *in vivo* 2 h after i.p. injection of α-GalCer. Data are from three experiments where three mice aged 5- to 6-week old were used per experiment.

Figure S4 Unchanged cytokine production and Fas-L expression by spleen invariant natural killer T cell (iNKT) cells in the absence of NK group 2 member D upon concanavalin A (Con A) administration. Representative intracellular staining of interferon (IFN)-γ, interleukin IL-4, and TNF-α production **(A)**, or FAS-L expression **(B)** by spleen NK1.1^+^ iNKT after 2 h of i.v. Con A administration (15 mg/kg). The frequencies of positive cells and in addition mean florescence intensity (MFI) for FAS-L are shown in the histograms. Results are from three experiments where three mice of each genotype were used per experiment and presented as mean ± SD. Numbers represent percentages. Significance was evaluated with non-parametric Mann–Whitney *U* test.

Figure S5 Cytokine production and Fas-L expression by liver invariant natural killer T cell (iNKT) cells is not induced directly *in vitro* by concanavalin A (Con A). **(A)** Representative intracellular staining of interferon (IFN)-γ, interleukin IL-4, and TNF-α production expression by spleen NK1.1^+^ iNKT after 4 h incubation in the presence of Con A (10 μg/ml) or PMA/ionomycin. The frequencies of positive cells are shown in the histograms. **(B)** Cytokine level in the supernatant by spleen cells after O/N incubation with Con A or PMA/ionomycin at the previously indicated concentration. **(C)** Representative cell-surface staining of FAS-L expression by spleen NK1.1^+^ iNKT after 4 or 18 h incubation in the presence of Con A (10 μg/ml). Results are from three to four experiments where three mice of each genotype were used per experiment and presented as mean ± SD.

Figure S6 Model of the role of NK group 2 member D (NKG2D) expressed on invariant natural killer T cell (iNKT) cells in concanavalin A (Con A)-induced hepatitis. In wild-type mice, upon Con A administration: hepatocytes upregulate NKG2D-L cell-surface expression including retinoic acid early inducible 1 (RAE-1) (1); NKG2D-L interact with NKG2D constitutively expressed by liver iNKT cells (2); NKG2D signal iNKT cells to produce cytokines (3), and to express FAS-L (4); liver damage is caused by iNKT cell FAS–FAS-L mediated killing of hepatocytes and directly or indirectly by the cytokine produced by these cells (5). The absence of NKG2D in *Klrk1*^−/−^ mice shut off the aforementioned cascade of event reducing FAS-L expression and cytokine production leading to reduced iNKT cell cytotoxicity and liver injury and consequently increasing mice survival upon Con A administration.

## Abbreviations

IFN, interferon; IL, interleukin; iNKT, cell invariant natural killer T cell; *Klrk1*, killer cell lectin-like receptor subfamily K, member 1; NKG2D, NK group 2 member D; Con A, Concanavalin A; α-GalCer, α-galactosylceramide; Poly(I:C), Polyinosinic: polycytidylic acid; D-GalN, D-galactosamine.
