# Supplementary figures and images for: Critical Contribution of NK Group 2 Member D Expressed on Invariant Natural Killer T Cells in Concanavalin A-Induced Liver Hepatitis in Mice
Source: Front Immunol. 2018 May 17;9:1052. doi: 10.3389/fimmu.2018.01052 (PMC5966527; doi:10.3389/fimmu.2018.01052)

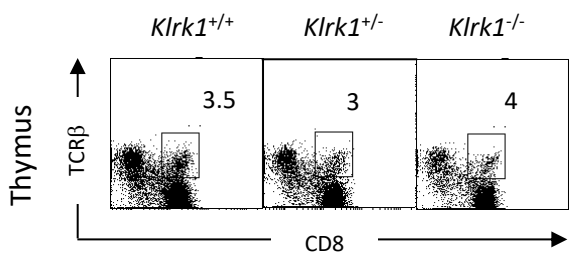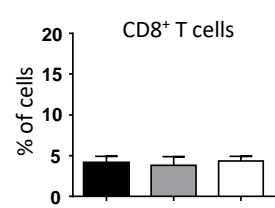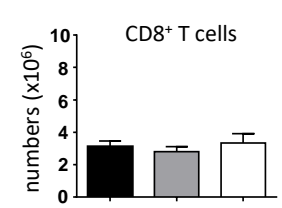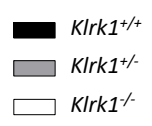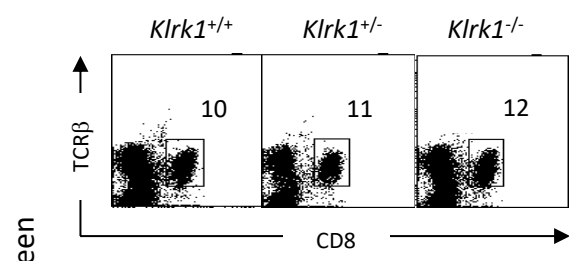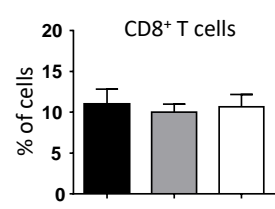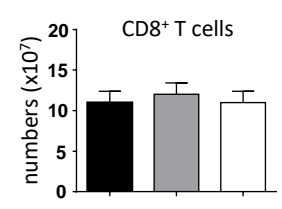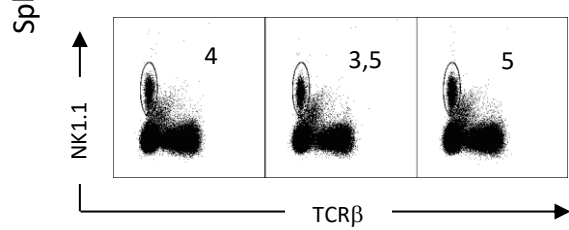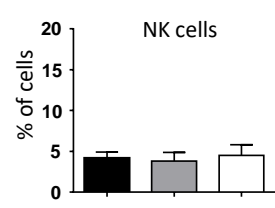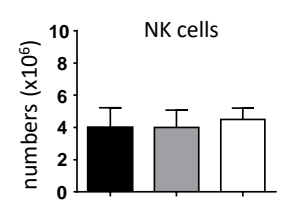

Supplement: Figure S1 — Frequency and numbers of CD8+ and NK cells in the absence of NK group 2 member D. Representative Flow cytometry analysis of CD8/TCRβ and NK1.1/TCRβ staining in the thymus and spleen. Histograms show frequencies of CD8+ cells (CD8+/TCRβ+) and NK cells (NK1.1+/TCRβ−) as defined by the gates. Data are from five experiments where three mice aged 5- to 6-week were used per experiment and presented as mean ± SD. Numbers represent percentages. [file Image_1.PDF]

A

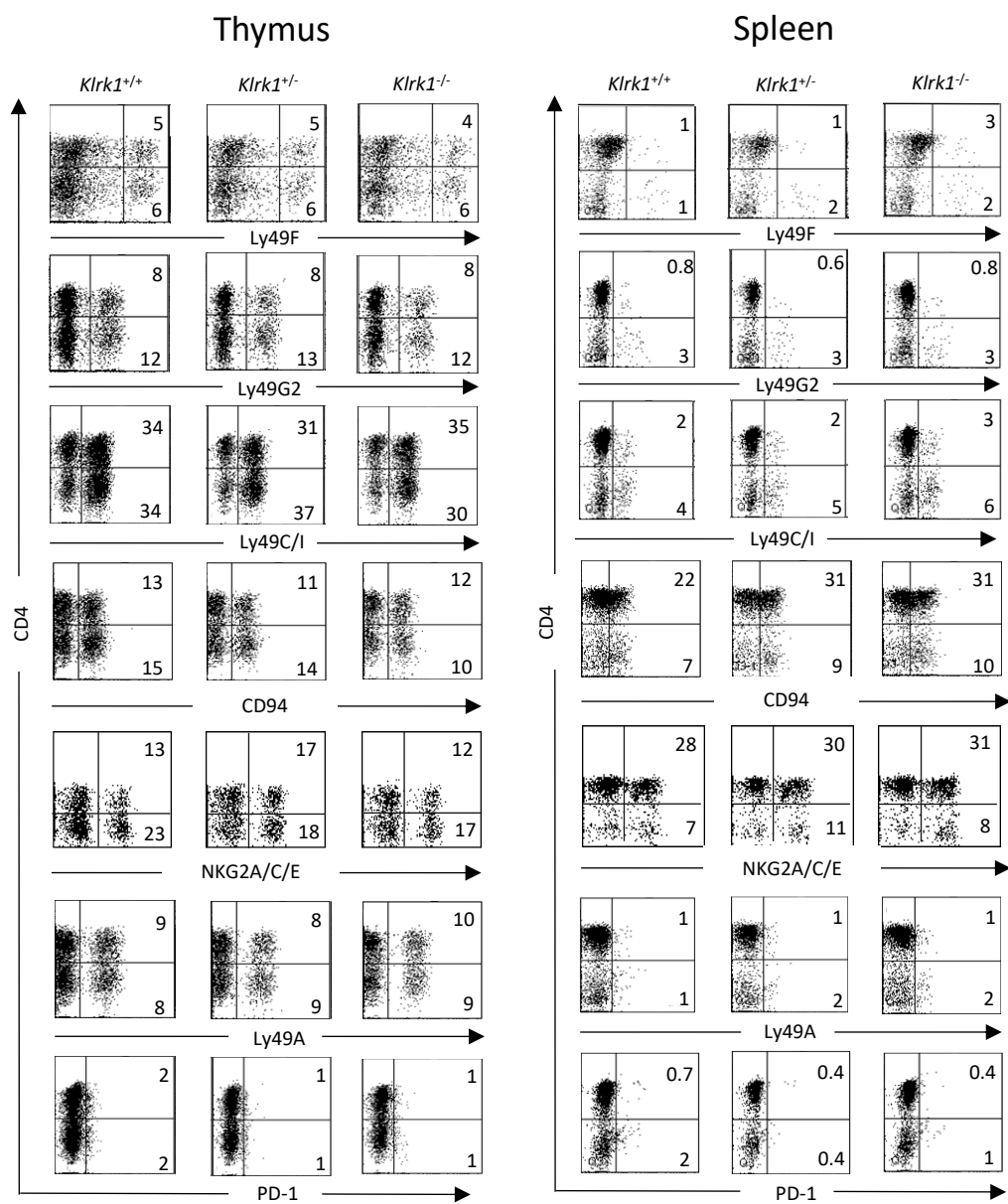

B

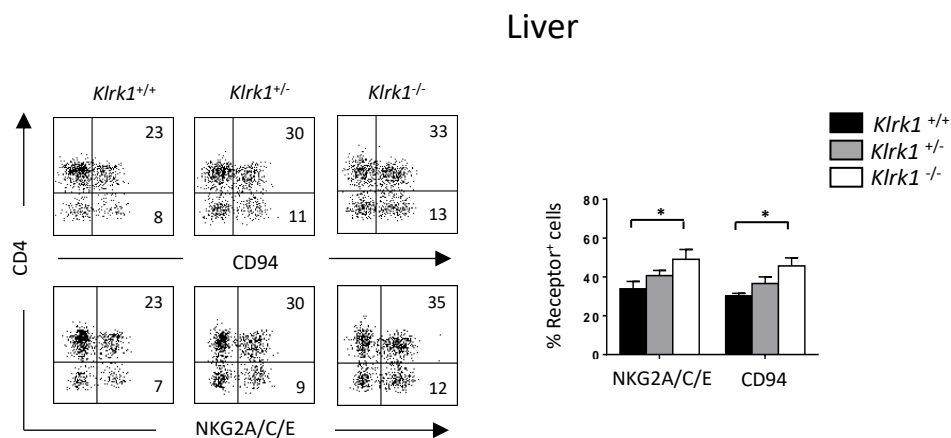

C

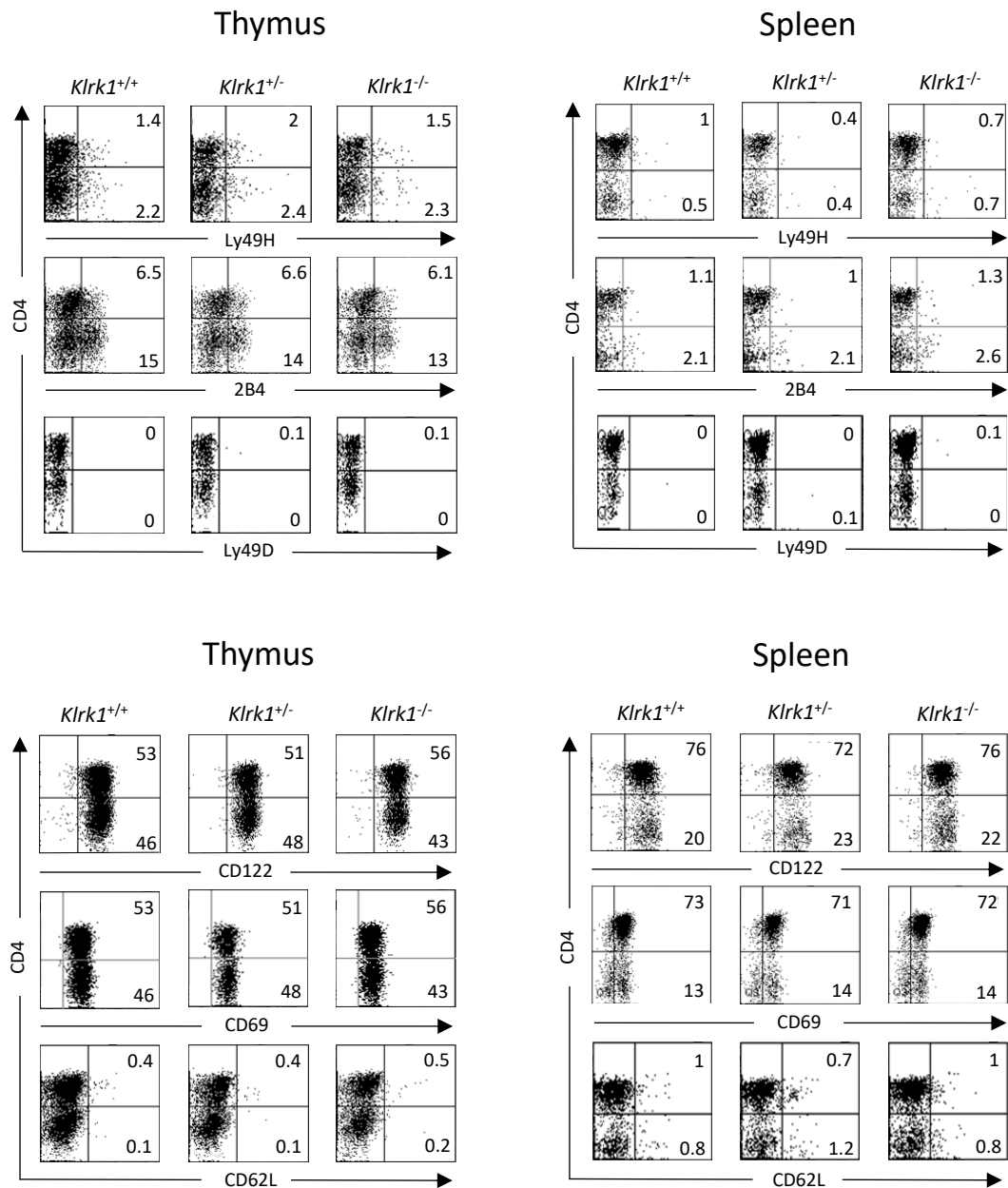

Supplement: Figure S2 — Phenotypic characterization of invariant natural killer T cell (iNKT) cells in the absence of NK group 2 member D. (A). Representative staining of inhibitory NK-receptors among NK1.1+ iNKT cells in the thymus and spleen. (B) Representative staining of CD94 and NKG2A/C/E inhibitory NK-receptors among NK1.1+ iNKT cells in the liver. Histograms show frequencies. (C) Representative staining of activating NK-receptors (upper panel) and other activating receptors (lower panel) among NK1.1+ iNKT cells in the thymus and spleen. Numbers represent percentages. Data are representative of five experiments where three mice aged 5- to 6-week old were used per experiment and presented in histograms as mean ± SD. Significance is represented by an asterisk and was evaluated with non-parametric Mann–Whitney U test. [file Image_2.PDF]

A

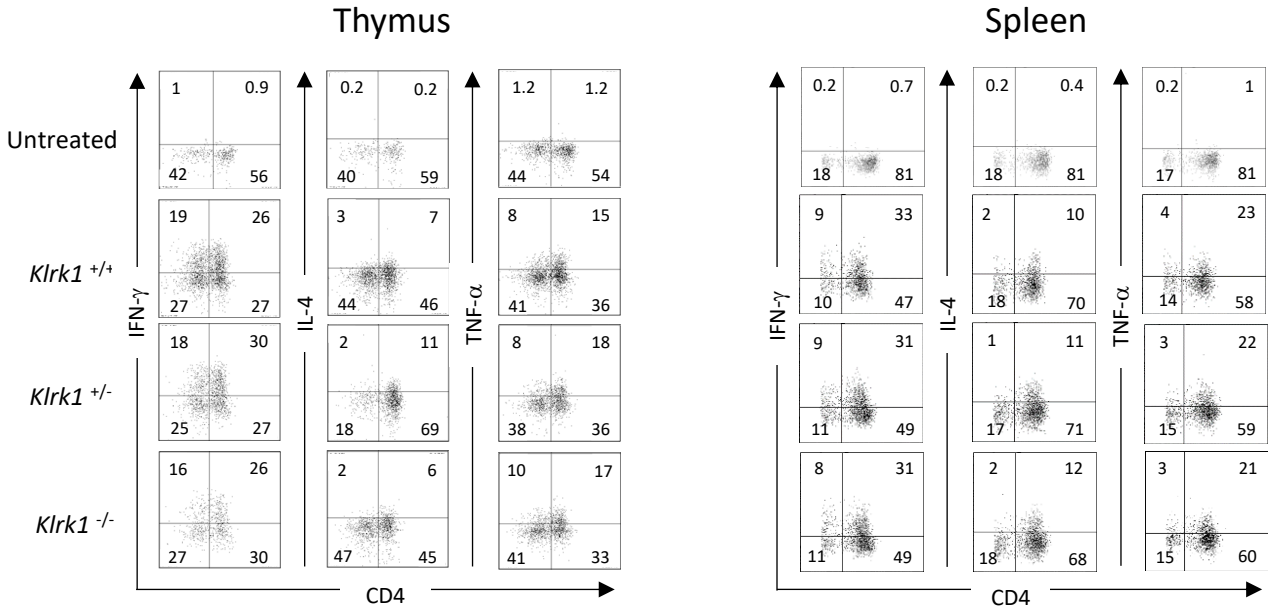

B

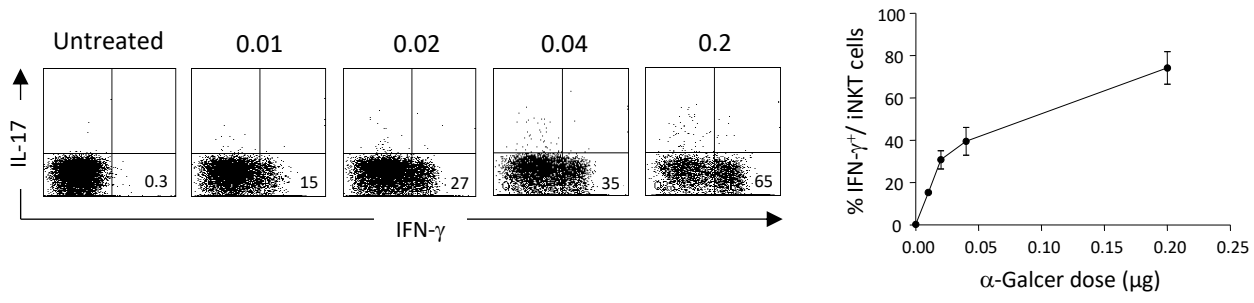

C

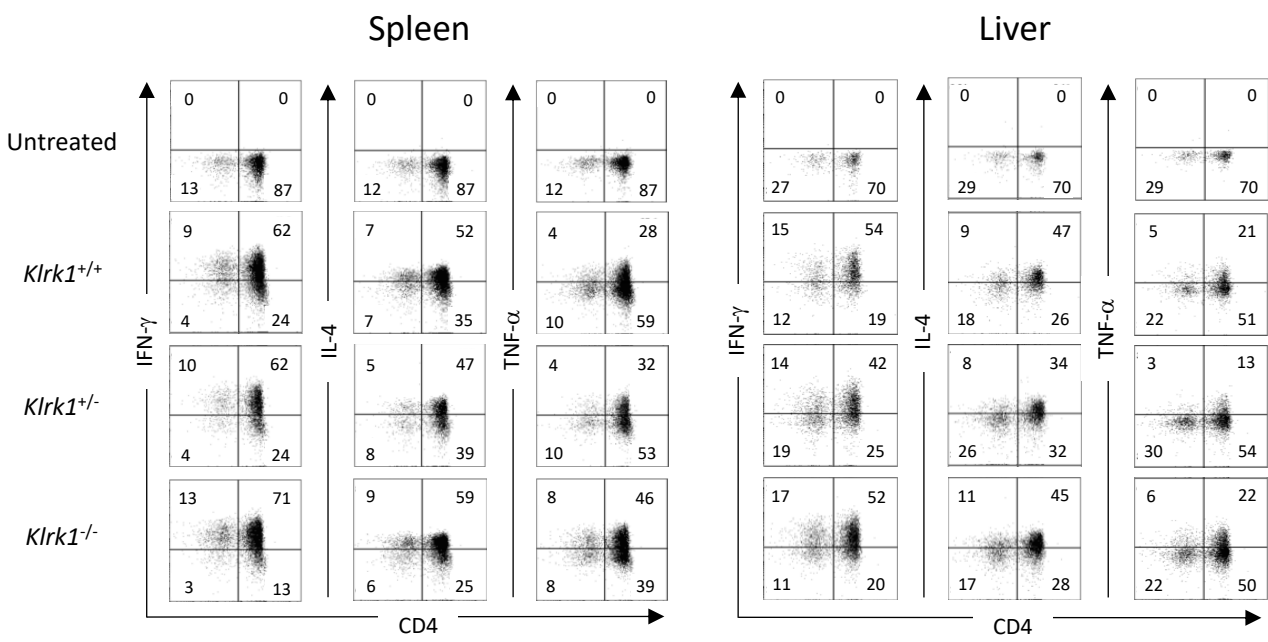

Supplement: Figure S3 — Cytokine production capabilities of invariant natural killer T cell (iNKT) cells in the absence of NK group 2 member D. (A) Representative staining of interferon (IFN)-γ, interleukin (IL)-4, and TNF-α production by iNKT cells in vitro, assessed by intracellular staining, in the thymus and spleen upon PMA/ionomycin stimulation for 4 h. (B) IFN-γ production by spleen iNKT cells assessed by intracellular staining in response to increased doses of α-galactosylceramide (α-GalCer) injected i.p. in normal B6 mice. (C) Representative staining of IFN-γ, IL-4, and TNF-α production by iNKT cells in the spleen and liver in vivo 2 h after i.p. injection of α-GalCer. Data are from three experiments where three mice aged 5- to 6-week old were used per experiment. [file Image_3.PDF]

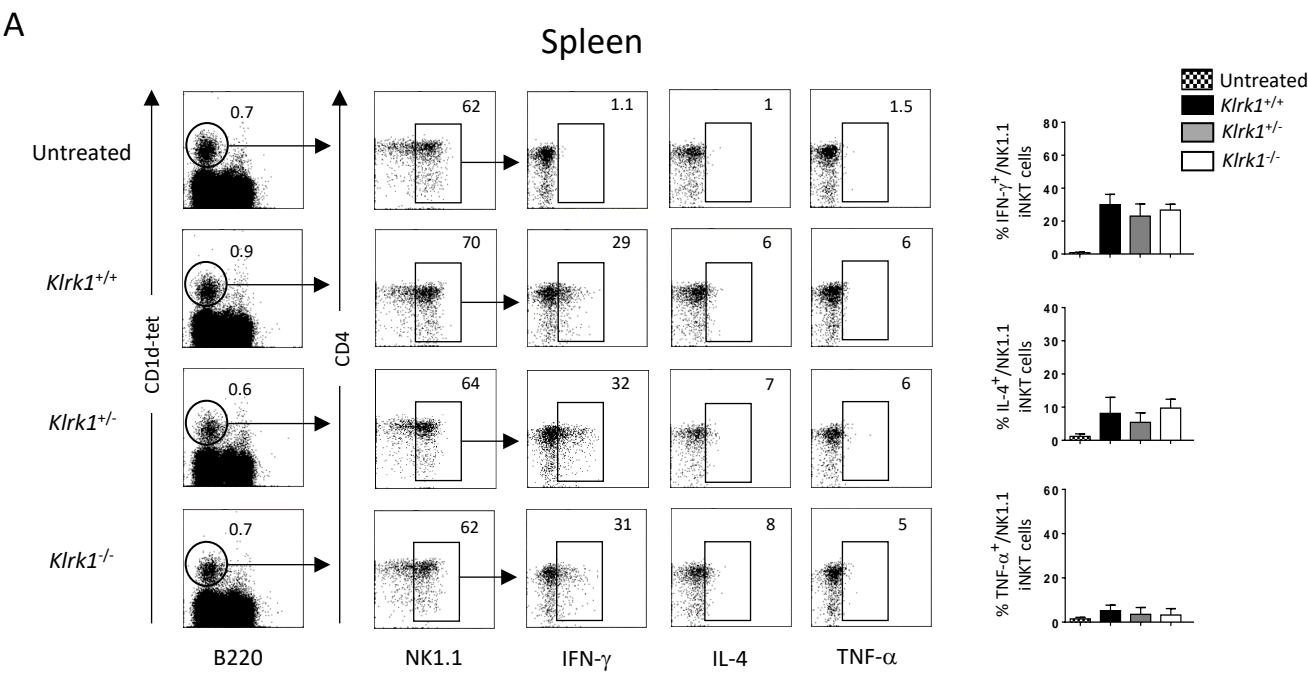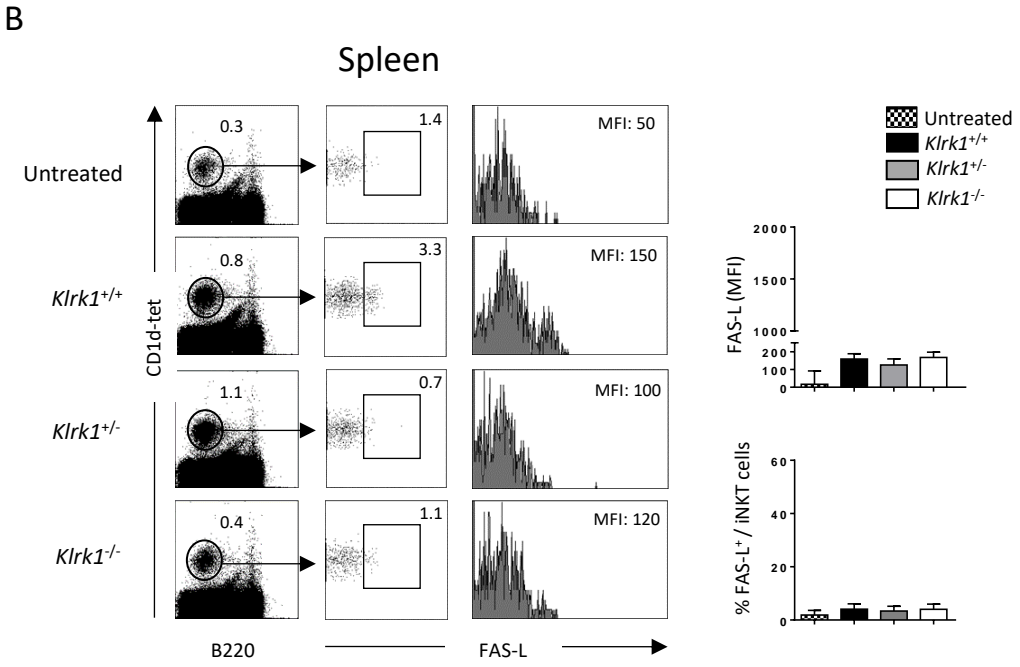

Supplement: Figure S4 — Unchanged cytokine production and Fas-L expression by spleen invariant natural killer T cell (iNKT) cells in the absence of NK group 2 member D upon concanavalin A (Con A) administration. Representative intracellular staining of interferon (IFN)-γ, interleukin IL-4, and TNF-α production (A), or FAS-L expression (B) by spleen NK1.1+ iNKT after 2 h of i.v. Con A administration (15 mg/kg). The frequencies of positive cells and in addition mean florescence intensity (MFI) for FAS-L are shown in the histograms. Results are from three experiments where three mice of each genotype were used per experiment and presented as mean ± SD. Numbers represent percentages. Significance was evaluated with non-parametric Mann–Whitney U test. [file Image_4.PDF]

A

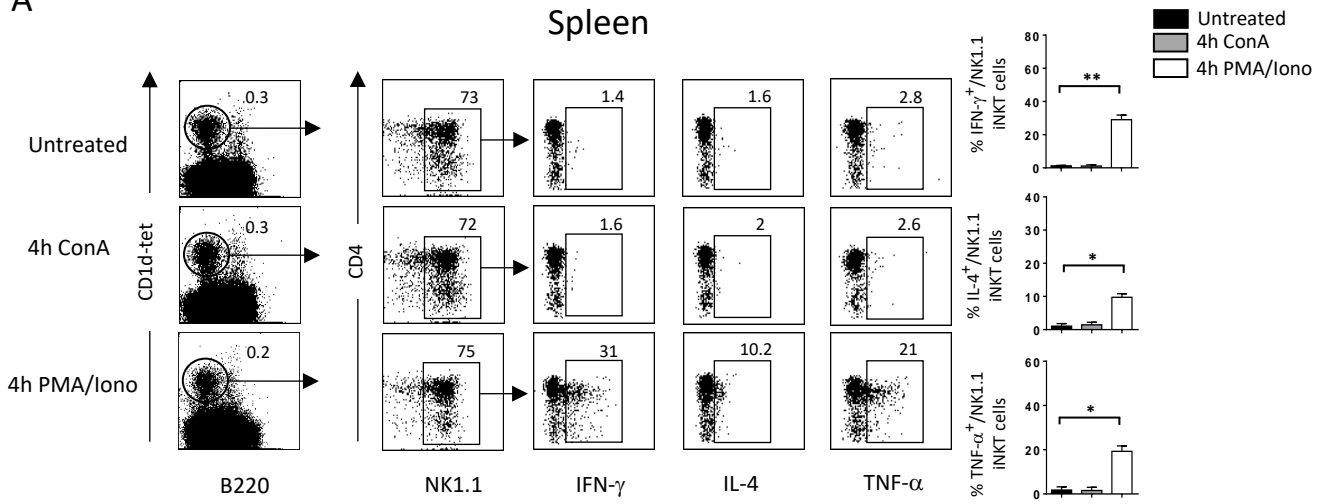

B

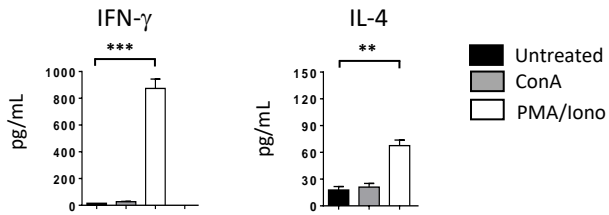

C

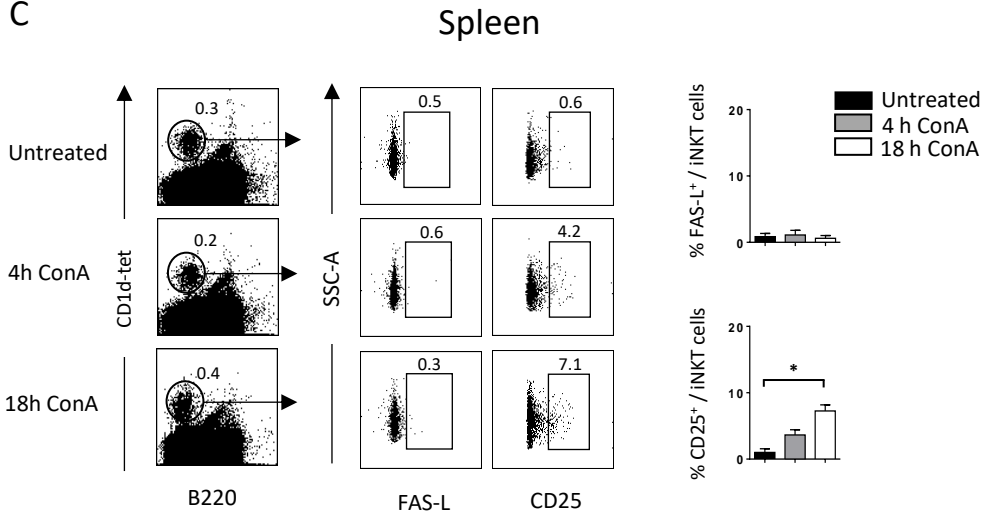

Supplement: Figure S5 — Cytokine production and Fas-L expression by liver invariant natural killer T cell (iNKT) cells is not induced directly in vitro by concanavalin A (Con A). (A) Representative intracellular staining of interferon (IFN)-γ, interleukin IL-4, and TNF-α production expression by spleen NK1.1+ iNKT after 4 h incubation in the presence of Con A (10 μg/ml) or PMA/ionomycin. The frequencies of positive cells are shown in the histograms. (B) Cytokine level in the supernatant by spleen cells after O/N incubation with Con A or PMA/ionomycin at the previously indicated concentration. (C) Representative cell-surface staining of FAS-L expression by spleen NK1.1+ iNKT after 4 or 18 h incubation in the presence of Con A (10 μg/ml). Results are from three to four experiments where three mice of each genotype were used per experiment and presented as mean ± SD. [file Image_5.PDF]

*Klrk1*<sup>+/+</sup>

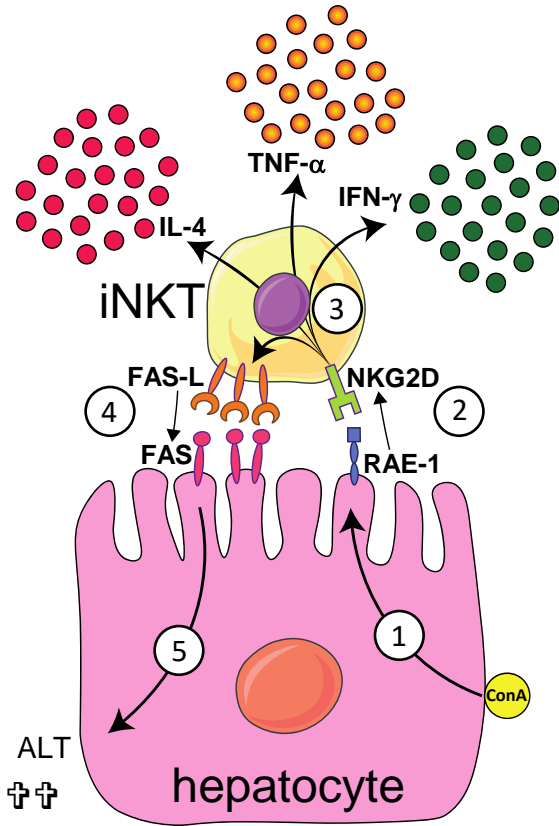

*Klrk1*<sup>-/-</sup>

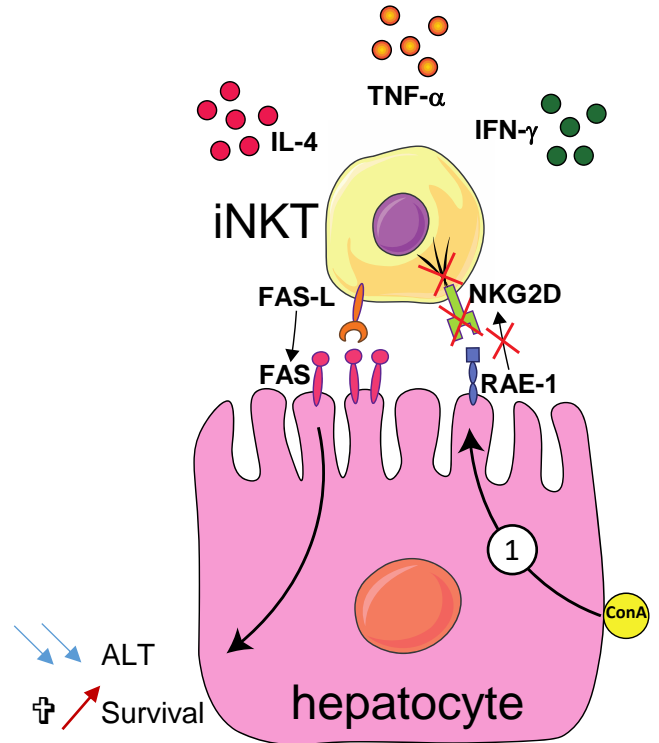

Supplement: Figure S6 — Model of the role of NK group 2 member D (NKG2D) expressed on invariant natural killer T cell (iNKT) cells in concanavalin A (Con A)-induced hepatitis. In wild-type mice, upon Con A administration: hepatocytes upregulate NKG2D-L cell-surface expression including retinoic acid early inducible 1 (RAE-1) (1); NKG2D-L interact with NKG2D constitutively expressed by liver iNKT cells (2); NKG2D signal iNKT cells to produce cytokines (3), and to express FAS-L (4); liver damage is caused by iNKT cell FAS–FAS-L mediated killing of hepatocytes and directly or indirectly by the cytokine produced by these cells (5). The absence of NKG2D in Klrk1−/− mice shut off the aforementioned cascade of event reducing FAS-L expression and cytokine production leading to reduced iNKT cell cytotoxicity and liver injury and consequently increasing mice survival upon Con A administration. [file Image_6.PDF]
